# Poly(Ethylene Glycol)–Polylactide Micelles for Cancer Therapy

**DOI:** 10.3389/fphar.2018.00202

**Published:** 2018-03-08

**Authors:** Jixue Wang, Shengxian Li, Yuping Han, Jingjing Guan, Shirley Chung, Chunxi Wang, Di Li

**Affiliations:** ^1^Department of Urology, The First Hospital of Jilin University, Changchun, China; ^2^Key Laboratory of Polymer Ecomaterials, Changchun Institute of Applied Chemistry, Chinese Academy of Sciences, Changchun, China; ^3^Department of Urology, China-Japan Union Hospital of Jilin University, Changchun, China; ^4^Department of Biology, University of Waterloo, Waterloo, ON, Canada

**Keywords:** polylactide, poly(ethylene glycol), micelle, nanocarrier, controlled drug release, antitumor treatment

## Abstract

For the treatment of malignancy, many therapeutic agents, including small molecules, photosensitizers, immunomodulators, proteins and genes, and so forth, have been loaded into nanocarriers for controllable cancer therapy. Among these nanocarriers, polymeric micelles have been considered as one of the most promising nanocarriers, some of which have already been applied in different stages of clinical trials. The successful advantages of polymeric micelles from bench to bedside are due to their special core/shell structures, which can carry specific drugs in certain disease conditions. Particularly, poly(ethylene glycol)–polylactide (PEG–PLA) micelles have been considered as one of the most promising platforms for drug delivery. The PEG shell effectively prevents the adsorption of proteins and phagocytes, thereby evidently extending the blood circulation period. Meanwhile, the hydrophobic PLA core can effectively encapsulate many therapeutic agents. This review summarizes recent advances in PEG–PLA micelles for the treatment of malignancy. In addition, future perspectives for the development of PEG–PLA micelles as drug delivery systems are also presented.

## Introduction

Cancer is one of the major health problems that threaten human life. According to the worldwide statistic, there were 14.1 million new tumor incidences and 8.2 million cancer-related deaths in 2012 (Scsukova et al., 2015). Cancer cells proliferate uncontrollably and rapidly, thus they are characterized by the development of abnormalities and the combination of mutagenic stages. Moreover, they can realize self-sufficiency in growth signals, resistance to growth inhibition and evasion of apoptotic cues (Luo et al., 2009). Furthermore, tumors can induce angiogenesis, evasion from immune surveillance, and metastasis to distant sites through interactions with surrounding stromal cells (Mohme et al., 2017). All of these reasons have led to the refractoriness of cancer. To date, chemotherapy, radiation, surgery, and hormonal therapies are still the major treatment methods for cancer in clinics. Whereas in the research industry, many other treatments, such as photodynamic therapy, photothermal therapy, gene therapy, immunotherapy, and so forth, are being studied. Despite the relatively satisfactory results these therapeutic agents exhibit, they also possess many disadvantages, including poor pharmacokinetics, unspecific bio-distribution, and low targeting ability. The poor solubility and hydrophobicity are considered the major hurdles when therapeutic agents are applied in cancer therapy. Therefore, it is urgent and necessary to overcome these shortcomings to enhance the anti-tumor efficiency.

With the development of nanotechnology, nanomaterials have been widely used in biological application, such as biosensor, tissue engineering as well as drug delivery (Yu et al., 2016a,b,c; Zhang et al., 2017). Nanocarriers have attracted more and more attention in cancer therapy owing to their unique properties, such as nanoscaled size, high surface-to-volume ratio, and favorable physico-chemical characteristics. Various nanocarriers, including liposomes, micelles, and nanocapsules, have been studied in anticancer trials (He et al., 2016; Hofmann et al., 2017; Niu et al., 2017; Tao et al., 2017). They have the capacity to modulate both pharmacokinetic and pharmacodynamic properties, thereby improving their therapeutic index. Among these nanocarriers, polymeric micelles have gained considerably more attention as a multifunctional drug delivery system for poorly water-soluble agents.

Polymeric micelles are the nano-scaled sized particles (5–200 nm) which are self-assembled by amphiphilic polymers. They consist of two parts: the hydrophobic part on the inside (core) and hydrophilic part on the outside (shell). Therefore, the hydrophobic core can serve as a solubilization depot for agents with poor aqueous solubility. The hydrophilic shell provides advantages including longer blood circulation time and increased stability in the blood. In addition, polymeric micelles can be functionalized with targeting ligands to enhance tumor accumulation. As a result, the role of polymeric micelles in delivery of hydrophobic therapeutic agents for anticancer therapy is promising and opportunistic.

Polyethylene glycol (PEG)-polylactide (PLA) is one of the most prominent amphipathic polymers, therefore it is very suitable for constructing micelles. PLA is a form of biodegradable and biocompatible polyester derived from renewable resources and approved by the Food and Drug Administration (FDA) for clinical use. The hydrophobicity of PLA makes it suitable for the hydrophobic portion of micelles. PLA has three types of stereoisomers: poly(L-(-)-*S*-lactide) (PLLA), poly(D-(+)-*R*-lactide) (PDLA), and racemic PDLLA. Interestingly, PLLA and PDLA can form stereocomplexes through physical association of PLLA and PDLA chains (Ikada et al., 1987). PLA can interact with different hydrophilic agents, such as PEG (Danafar et al., 2017), poly(2-methacryloyloxyethyl phosphorylcholine) (PMPC) (Long et al., 2016), poly(ethylene oxide) (PEO) (Fang et al., 2015), and poly(*N*-isopropylacrylamide) (PNIPAAm) (Wei et al., 2009), to form amphiphilic block copolymers and further self-assemble into micelles. Among them, PEG is the most popular hydrophilic agent due to its various advantages, including linearity, lack of charge, immunogenicity, low polydispersity, and easy activation for conjugation. PEG–PLA micelles have been widely used as drug delivery systems for cancer therapy owing to the excellent physicochemical and biological properties, namely nontoxicity, non-protein adsorption, and weakened uptake by the reticulo-endothelial system (RES) after intravenous injection (Wang et al., 2015, 2017). Notably, Genexol®-PM, a paclitaxel formulation based on PEG–PLA copolymer micelles, was approved in Korea in 2007 for the treatment of breast, lung, and ovarian cancers (Luo et al., 2012). Moreover, it is currently under clinical development in the USA (Lee et al., 2008).

**Scheme 1:**
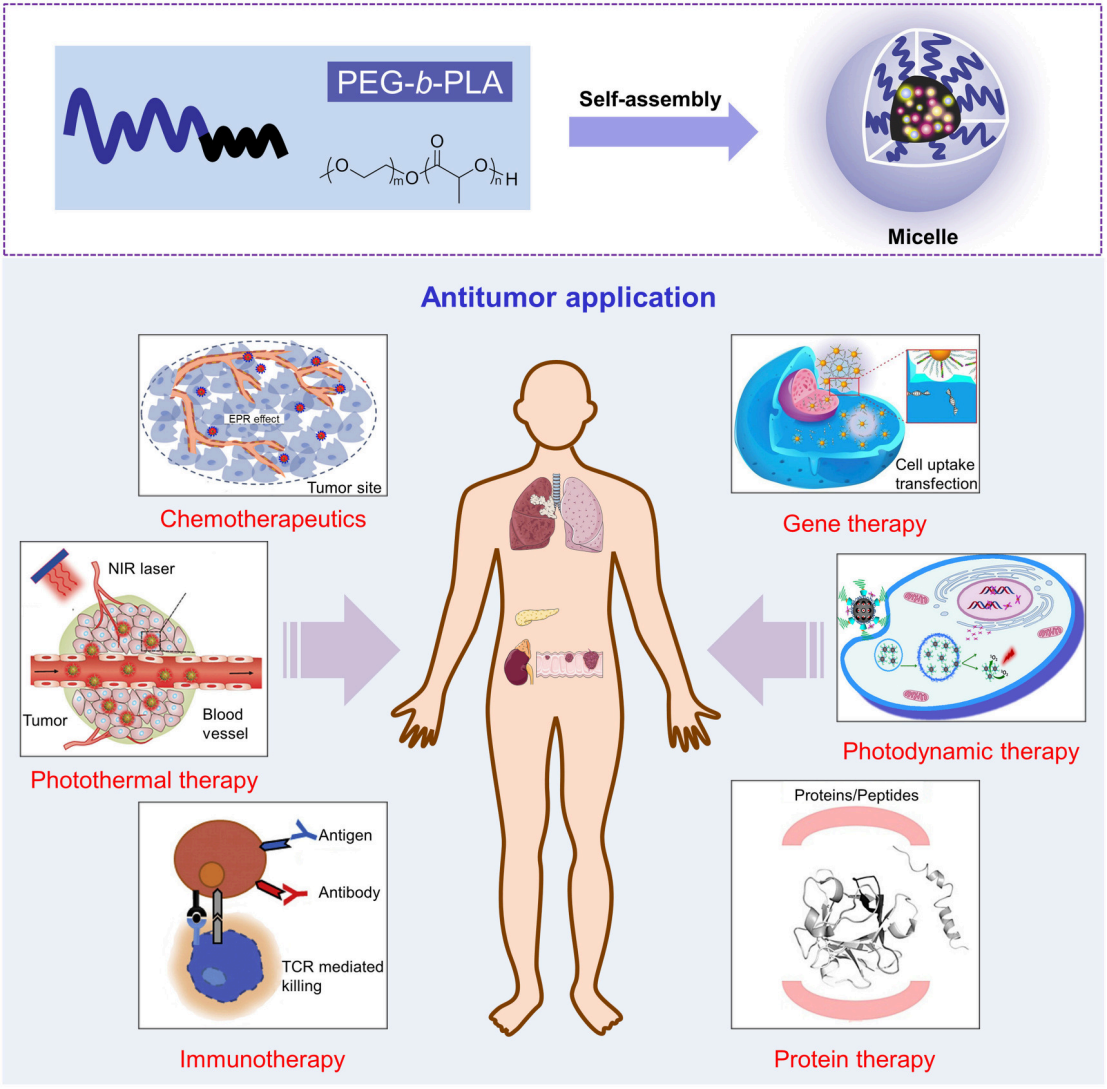
Schematic illustration of PEG-*b*-PLA micelle in application of antitumor. Reproduced with permission from Cho et al. (2016); Peng et al. (2016); Wang et al. (2016); Moy and Tunnell (2017); Yin et al. (2017) and Zhai et al. (2017).

In this review paper, an overview of PEG–PLA-based micelles utilized for the effective delivery of therapeutic agents possessing varying mechanisms for cancer treatment is discussed, as shown in Scheme 1. Additionally, the applications of different treatment modalities are described in detail. Features of nanocarriers in references are shown in Table 1. In particular, the development of stimuli-responsive, targeted-modified, and multifunctional PEG–PLA micelles are also highlighted.

**Table 1:** Features of nanocarriers in references.

**Treatment methods**	**Therapeutic agents**	**Mean size (nm)**	**Delivery strategy**	**Cancerous cells**	**References**
Chemotherapeutics	AG 050	10–100 nm	EPR	KKU-M213 cells	Puntawee et al., 2016
	DTX	58.2 ± 2.3 nm	EPR	HSC-3 cells	Shi et al., 2016
		30–230 nm		KB cells	Yu and Qiu, 2016
		111 nm/129 nm	c(RGDfK) targeted	HeLa cells	Li et al., 2016a
		~ 80 nm	Octreotide targeted	NCI-H446 cells	Zhang et al., 2011
	PTX	80–125 nm	Folate targeted	KB cells	Xiong et al., 2011
		14.6 ± 0.8–104.2 ± 8.1 nm	pH sensitive/EPR	A549 cells	Liang et al., 2016
	DOX	170.87 ± 3.02	Folate targeted/pH sensitive	MCF-7Adr	Li et al., 2015
		150 nm	pH sensitive	MDA-MB231	Wu et al., 2010
		34–107 nm	Redox-responsive	HeLa cells	Yang et al., 2015
Photothermal therapy	TPT–TT NPs	85 nm	Optical excitation	HeLa/HepG2 cells	Sun et al., 2015
Photodynamic therapy	PpIX	80 nm	Optical excitation	C26/B16BL6/Lewis cells	Ogawara et al., 2016
		30 nm	Optical excitation	H2009 cells	Ding et al., 2011a
		49 ± 6/57 ± 6 nm	Optical excitation	H2009 cells	Ding et al., 2011b
	NEt2Br2BDP	138.4 ± 17.3 nm	Optical excitation/pH sensitive	U87MG cells	Tian et al., 2015
Immune therapy	LD-indolicidin	25 ± 5 nm		EG7 cells	Coumes et al., 2015
	CTLA-4-siRNA	141.6 ± 6.1 nm		B16 melanoma cells	Li et al., 2016b
Protein therapy	Plk1; si*Plk1*	120 nm	ScFv_Her2_ targeted	BT474	Dou et al., 2014
	OX26	50 nm	OX26 targeted		Yue et al., 2012
Gene therapy	TNF cDNA	80 ± 4 nm	EPR	MCF-7 cells	Shukla et al., 2017
	siRNA	54.30 ± 3.48 nm	EPR	MCF-7 cells	Zhao et al., 2012
	TRAIL gene/PTX		RGD targeted	U87 cells	Zhan et al., 2012
Others	curcumin	~ 33 ± 2.3 nm	EPR	C6/U251 cells	Zheng et al., 2016
		171.0–22.6 nm	pH sensitive/EPR	MCF-7 cells	Yu et al., 2014
		104.6 ± 2.1/169.3 ± 1.52 nm	EPR	B16F10/MDA-MB-231 cells	Kumari et al., 2017
		<100 nm	EPR	HepG2 cells	Yang et al., 2012c
		110 ± 5 nm	EPR	B16F10/MDA-MB-231 cells	Kumari et al., 2016
	DOX/CA4	29.2 ± 2.5 nm	EPR	B16-F10	Wang et al., 2011

## Chemotherapeutics

In clinical settings, surgery and radiotherapy are the most commonly used and effective therapeutic means for local and non-metastatic tumors. However, they are inefficient for metastatic tumors. Currently, application of anti-cancer drugs, such as chemotherapeutic drugs, hormone drugs, and biological drugs, has become the main method of treatment. These anti-cancer drugs are able to reach all parts of the body *via* the bloodstream and primarily inhibit the rapid replication of tumor cells. Unfortunately, they also inhibit the rapid growth of healthy cells which are crucial to maintaining normal function of the organism, such as hair follicles, bone marrow, and gastrointestinal tract cells (Chabner and Roberts, 2005). As a result, serious side effects are apparent in chemotherapeutic drug treatments. Due to the severe toxicity and concomitant multidrug resistance of conventional chemotherapeutic drugs, it is urgent to find new effective drug carriers to solve these problems. PEG–PLA micelles provide significant advantages over standard treatments. Drug-loaded micelles exhibit a huge potential for tumor drug delivery to overcome the limitations of chemotherapeutic agents.

As generally known, many chemotherapeutic drugs are not highly water-soluble. Hydrophobic agents are associated with several problems in therapeutic applications, such as poor absorption, bioavailability, and drug aggregation-related complications. Fortunately, polymeric micelles constructed from amphiphilic copolymers can promisingly increase the water solubility of such hydrophobic chemotherapeutic drugs by 10–5000 folds (Savić et al., 2006). Puntawee et al. improved the aqueous solubility and bioavailability of semi-synthetic andrographolide analog (19-triphenylmethyl ether andrographolide, AG 050) by utilizing the PEG-*b*-PLA micelles. As a result, PEG-*b*-PLA micelle was able to significantly increase the encapsulation efficiency of hydrochloride salt of AG 050 (AG 050-P) in aqueous solution (280-fold) (Puntawee et al., 2016).

**Figure 1:**
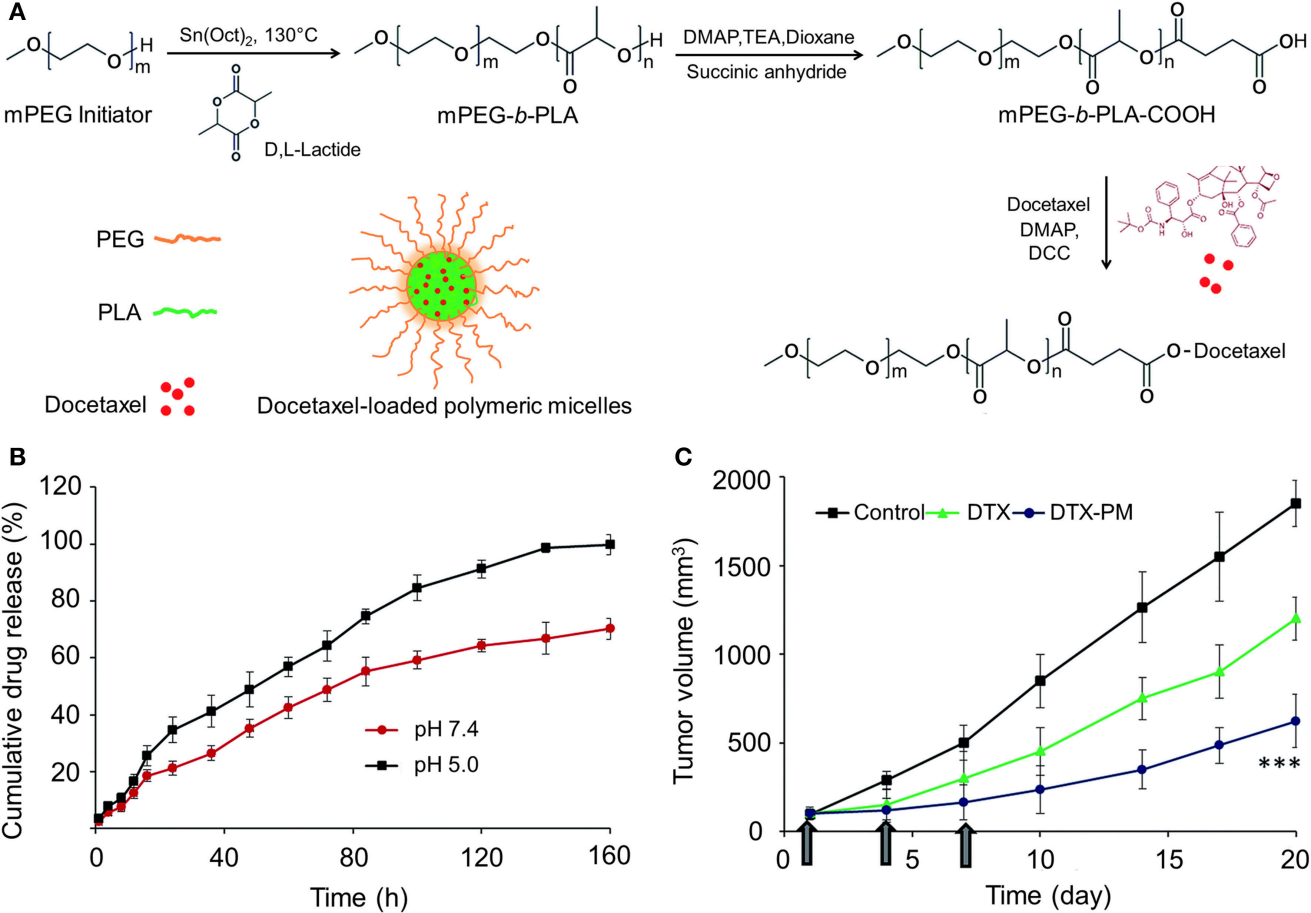
**(A)** Schematic illustration of synthesis of mPEG–PLA-docetaxel polymer drug conjugate. **(B)** The *in vitro* release of DTX from DTX-PM (PBS, pH 7.4 and pH 5.0, at 37 °C). **(C)** Tumor volume of free DTX and DTX-PM in xenograft tumor model (^***^P < 0.0001). Reproduced with permission from Shi et al. (2016).

It is worth noting that conventional chemotherapeutic drugs are quite small in size. As a result, they are rapidly cleared from the bloodstream, thus leading to decreased concentration within the tumor (Allen and Cullis, 2004). When chemotherapeutic drugs are loaded into PEG–PLA micelles, their circulation time in the bloodstream will be prolonged, therefore allowing adequate amounts of drugs to reach the target site (Jin et al., 2017). Furthermore, compared to other drug carriers, micelles have the advantage of possessing a very small size (10–100 nm). This is critical for passive accumulation through the leaky vasculature and into the tumor site *via* the enhanced permeability and retention (EPR) effect to solid tumors, particularly to poorly vascularized tumors (Davis and Shin, 2008; Feng et al., 2017). Two main factors are responsible for the EPR effect: (1) the angiogenic vasculature of tumor sites have higher permeability than normal vasculature because of their discontinuous endothelia and (2) the lymphatic drainage is not fully developed in tumors. These conditions result in the exudation of colloidal particles through the “leaky” endothelial layer of tumor vascular and subsequently accumulating in tumor tissues. Majority of vascular pore cutoff size of tumor sites is between 380 and 780 nm (Hobbs et al., 1998). Shi et al. synthesized a docetaxel (DTX)-conjugated mPEG–PLA micelle *via* an ester linkage (DTX-PM). The average size of DTX-PM was 58.2 ± 2.3 nm, which was suitable for passive targeting *via* an EPR effect for anti-cancer drug delivery. More importantly, the DTX-PM significantly induced apoptosis of HSC-3 cancer cells and effectively suppressed the tumor progression in the HSC-3 xenograft model *in vivo* (Shi et al., 2016) (Figure 1). Particle size is a critical factor in the efficacy of drug-loaded-micelles. It is of fundamental importance to understand the association between the size of drug-loaded nanocarriers and their fate in biological systems *in vivo* for rational design of drug delivery systems. Yu et al. prepared five different sized (30–230 nm) DTX-loaded methoxy poly(ethylene glycol)-poly(lactide) micelles. They studied the permeation effect of micelles within both multicellular tumor spheroids and tumor xenografts in mice. The results indicated that small micelles could enhance the tissue penetration however, the blood half-life was short. Micelles of 100–200 nm showed prolonged circulation time compared with that of smaller or larger micelles, but the penetration was limited. Large micelles (>200 nm) had both short half-life and weak penetration ability, leading to limited tumor accumulation (Yu and Qiu, 2016). Through the EPR effect, drug-loaded PEG–PLA micelles can selectively kill tumor cells and reduce the damage to normal tissues.

Active targeting is another effective method to reduce the damage of chemotherapeutic drugs to normal tissues (Allen, 2002; Szakács et al., 2006). Ligands are modified on the surface of nanocarriers to bind to particular receptors overexpressed by tumor cells or tumor vasculature. Many kinds of targeted ligands have been researched, such as small organic molecules, sugar moieties, peptides, and monoclonal antibodies (Allen, 2002; Brannon-Peppas and Blanchette, 2012). Folate is an example of a small organic molecule that is a cancer targeting ligand because the folate receptor is frequently overexpressed on tumors (Cho et al., 2016). Xiong *et al*. designed folate-conjugated interfacially crosslinked biodegradable micelles consisting of poly(ethylene glycol)-*block*-poly(acryloyl carbonate)-*block*-poly(D,L-lactide) (PEG–PAC–PLA) and folate-PEG–PLA (FA-PEG–PLA) block copolymers for receptor-mediated delivery of paclitaxel (PTX). Remarkably, folate-decorated PTX-loaded crosslinked micelles displayed significantly higher toxicity to KB cells than free PTX. This is most likely due to their much more efficient cellular uptake through FA receptor-mediated endocytosis. Flow cytometry studies also showed that folate-decorated FITC-labeled crosslinked micelles were much more efficiently taken up by KB cells than that of controls without folate ligands. All these results indicated that ligand-conjugated interfacially crosslinked PEG–PLA micelles have great potential in targeted cancer therapy (Xiong et al., 2011). Peptides are another form of actively used ligands in anticancer drug delivery. Li et al. developed DTX-loaded target micelles (c(RGDfK)-PEG–PLA/PEG–PLA/DTX) that were able to targeted delivery DTX to the tumor cells. Cellular uptake and 3-(4,5-dimethylthiazol-2-yl)-2,5-diphenyltetrazolium bromide (MTT) studies revealed that the target micelles were more efficiently taken up by HeLa cells in the presence of c(RGDfK) and significantly improved the cytotoxicity compared to that of non-target micelles (Li et al., 2016a). In another study, researchers constructed somatostatin analog octreotide (OCT)-modified, DTX loaded PEG-*b*-PLA micelles that bind to somatostatin receptors (SSTRs) overexpressed on tumor cells. The results showed that OCT-PM-DTX enhanced intracellular delivery efficiency in cells and exhibited higher retardation of tumor growth *in vivo* (Zhang et al., 2011).

In another study, researchers synthesized a folate modified pH sensitive targeted polymeric micelle to reduce the systemic toxicity of doxorubicin (DOX) and to increase the antitumor efficacy in a multi-drug resistant tumor model (Li et al., 2015). A pH sensitive targeted strategy is derived from the pH of the tumor microenvironment which is slightly lower (6.5–7.2) compared to physiological pH (7.4) (Tannock and Rotin, 1989; Gerweck et al., 2006). The triggering mechanisms of most pH sensitive micelles relate to the endosome and release of the loaded drugs into the cytoplasm. This is because the pH significantly drops to 5.0–6.5 in the endosome and to 4.5 in primary and secondary lysosomes (Hubbell, 2003; Schmaljohann, 2006). Therefore, based on the pH of endosomes/lysosomes, pH sensitive PEG–PLA-based micelles loaded with chemotherapeutic drugs have been widely studied to improve the efficiency of cancer therapy. The major strategies used for inducing pH-sensitive behaviors are changing the charges in the micellar system and the dissociating pH-dependent drug binding linkers. Liang *et al*. constructed size shifting micelle nanoclusters (MNC) based on a cross-linked framework interspersed with PEG–PLA micelles. After being internalized into tumor cells, the framework of MNC became swollen and disintegrated within the acidic environment of lysosomes *via* the proton sponge effect of PEI resulting in the release of nanosized micelles for further penetration (Liang et al., 2016). Wu et al. fabricated tumor-targeted and pH-responsive polymeric micelles by mixing AP peptide (CRKRLDRN) conjugated PEG-poly(D,L-lactic acid) block copolymer (AP-PEG–PLA) into the pH-responsive micelles of methyl ether poly(ethylene glycol) (MPEG)-poly(-amino ester) (PAE) block copolymer (MPEG-PAE). This micelle showed a sharp pH-dependent micellization/demicellization transition at the acidic environment of the tumor. When loaded with DOX, the DOX-loaded micelles exhibited excellent anticancer therapeutic efficacy (Wu et al., 2010).

Redox-responsive nanocarriers are another captivating direction of research for effective intracellular anticancer drug release (Meng et al., 2009; Cheng et al., 2011). Unlike pH-sensitive strategies which usually release drugs in the endo/lysosomal compartments, redox-responsive strategies aim to disassemble and release drugs in the cytosol and cell nucleus where many chemotherapeutic drugs, such as DOX and PTX, elicit their therapeutic effects (Zhang et al., 2012; Chuan et al., 2014). Since the amounts of glutathione (GSH) tripeptide in the cytosol and cell nucleus (~2–10 mM) are confoundedly higher than that of extracellular fluids and circulation (~2–20 μM), the intracellular redox-response is exceedingly fast and efficient. Yang et al. constructed a type of redox-responsive micelle which could self-assemble from dynamic covalent PEG–PLA block copolymers that contained a double disulfide linkage in the backbone. The *in vitro* drug release analyses indicated that a reductive environment could result in triggered drug release profiles. A cytotoxicity assay of DOX-loaded micelles indicated higher cellular proliferation inhibition against HeLa cells pretreated with 10 mM GSH monoester (GSH-OEt) for 2 h than that of nonpretreated ones (Yang et al., 2015). In order to further improve the anticancer efficiency of chemotherapeutic drug-loaded PEG–PLA micelles, dual or multi-stimuli responsive micelles that respond to a combination of two or more signals mentioned above have also been developed.

## Photothermal therapy components

**Figure 2:**
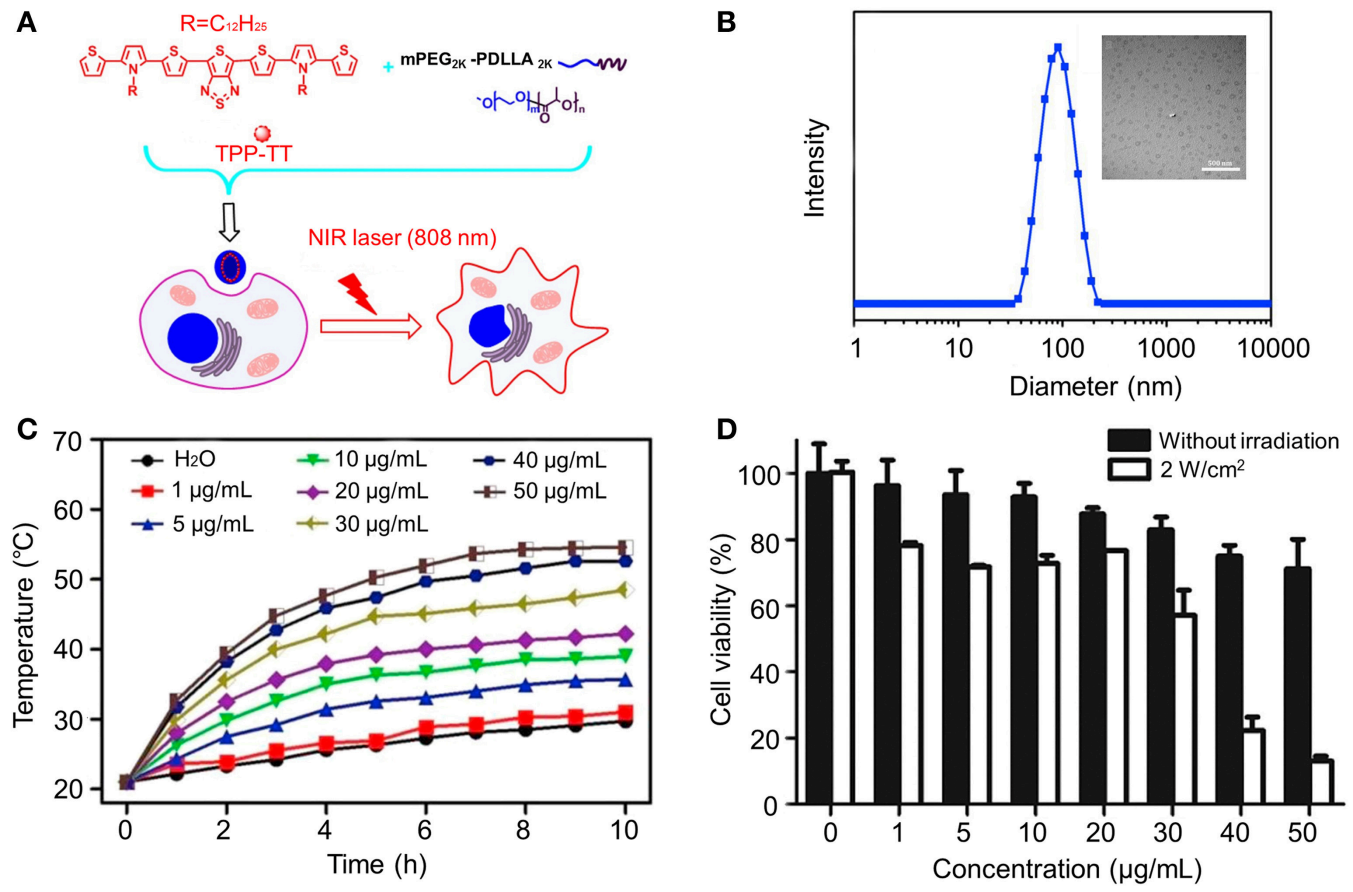
**(A)** Schematic illustration of the preparation and their cellular action process of TPT-TT NPs. **(B)** TEM image (Scale bar 500 nm) and size distribution determined by DLS of TPT-TT NPs. **(C)** Photothermal conversion behavior of TPT-TT NPs at various concentrations. **(D)** Relative cell viabilities of HepG2 cells incubated with different concentrations of TPT-TT NPs. Reproduced with permission from Sun et al. (2015).

Photothermal therapy (PTT) has become an effective alternative in cancer therapy because of the advantages over other treatment methods, such as minimal invasiveness, low toxicity, and high specificity to tumor sites (Geng et al., 2015; Mebrouk et al., 2015). In clinical practice, to maximize the light penetration depth and minimize the influence of biological chromophores, photothermal agents should have strong optical absorbance in the near-infrared (NIR) window (700–900 nm) (Jaque et al., 2014). In recent years, a variety of nanomaterials, such as gold nanostructures (nanoshells, nanorods, nanostars and nanocages) (Chen et al., 2010; Zhang et al., 2012; Ma et al., 2013; Wang et al., 2013), carbon nanomaterials (carbon nanotube and graphene) (Liu et al., 2011; Yang et al., 2012a), and various other inorganic (Tian et al., 2011) and organic nanoparticles (Yang et al., 2012b) have been used for PTT. Although most inorganic nanomaterials have shown effective therapeutic effect for cancers, majority of them are non-biodegradable and will retain in the body for a long time (Sharifi et al., 2012; Zhang et al., 2013). Compared to inorganic nanomaterials, polymeric NIR-absorbing nanomaterials agents have shown great superiority for PTT. Among PTT agents, small molecular organic dyes, such as (ICG) (Fang et al., 2017) and prussian blue (PB) (Hoffman et al., 2014), have been used not only as fluorescent probes in optical imaging, but also as PTT agents. Sun et al. fabricated a new photothermal nano-agent by coprecipitation of 2,5-Bis(2,5-*bis*(2-thienyl)-N-dodecyl pyrrole) thieno[3,4-b][1,2,5] thiadiazole (TPT-TT) and a biodegradable amphiphilic block copolymer, methoxy poly(ethylene glycol)2K-*block*-poly(D,L-lactide)2K (mPEG2K–PDLLA2K). As a result, TPT-NPs showed high photothermal conversion efficiency, excellent photostability, and heating reproducibility. The photostability of TPT-TT NPs was much better than that of ICG. Besides, TPT-TT NPs exhibited significant photothermal therapeutic effects toward human cervical carcinoma (HeLa) and human liver hepatocellular carcinoma HepG2 cells (Sun et al., 2015) (Figure 2).

## Photodynamic therapy components

Photodynamic therapy (PDT) is a light triggered method for cancer treatment. Due to the minimal aggressiveness and harmlessness to healthy tissue, PDT can avoid the disadvantages of conventional chemotherapeutic agents, such as serious side effects or multidrug resistances. PDT can kill tumor cells mainly through the generation of singlet oxygen (^1^O_2_) or free radicals. The reactive oxygen species (ROS) can cause significant cellular damage, destruction of tumor blood vessels, and stimulation of antineoplastic immunity (Juarranz et al., 2008). PDT depends on three factors to perform its anticancer effect: (i) a photosensitizer (PS) agent; (ii) irradiation of the affected region using light of an appropriate wavelength; and (iii) presence of oxygen. During irradiation, highly reactive species, such as ROS, which are capable of causing direct damage to biomolecules and triggering the death of tumor cells, are generated *in situ* (Paszko et al., 2011). Recently, the combination of photodynamic therapeutic agents and nanosystems has proven to be promising. Nanosystems, such as liposomes, micelles, and polymeric nanoparticles, have been widely studied for PDT application, as well as for combination of PDT agents with chemotherapeutic agents or with other forms of therapies. For example, Ogawara et al. encapsulated the photoprotoporphyrin IX dimethyl ester (PppIX-DME) into PEG–PLA nanoparticles (PN-Por). The PN-Por showed significant phototoxicity *in vitro* and effective antitumor effect in C26 tumor-bearing mice *in vivo*. All results exhibited the potency of PN-Por for PDT-based cancer treatments (Ogawara et al., 2016). In another study, in order to increase the solubility and delivery efficiency of PpIX, Ding et al. prepared PpIX loaded polymeric micelles using non-covalent encapsulation and covalent conjugation methods. Micelles with lower PpIX loading density (e.g., 0.2%) showed brighter fluorescence and higher ^1^O_2_ yield than higher PpIX loading density (e.g., 4%) in solution. However, 4% PpIX-conjugated micelles demonstrated better antitumor efficiency *in vivo* (Ding et al., 2011a). Moreover, they further studied the effect of carrier microenvironment on photophysical properties of 5,10,15,20-tetrakis(meso-hydroxyphenyl)porphyrin (mTHPP) and its biological efficacy in tumor inhibition. The electron-deficient PEG–PLA micelle was used as a control. Results displayed that the photophysical and photodynamic properties of mTHPP were highly related to the micelle core environment (Ding et al., 2011b).

**Figure 3:**
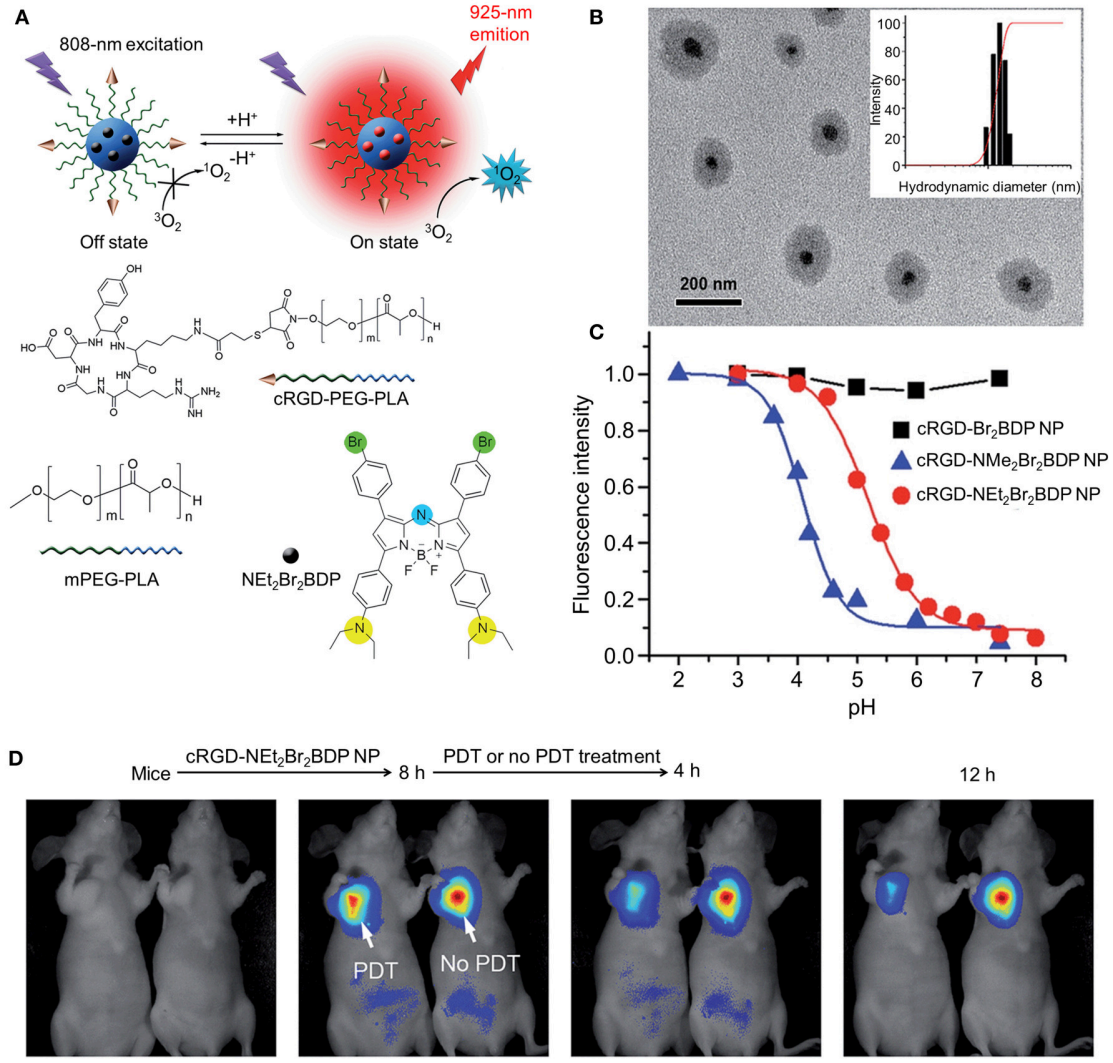
**(A)** Structure, characterization, and optical properties of cRGD-NEt2Br2BDP. **(B)** NPTEM image of cRGD-NEt_2_Br_2_BDP NP. Inset: size distribution of cRGD-NEt_2_Br_2_BDP NP determined with DLS. **(C)** pH titration curves of fluorescence intensity of different NPs. **(D)** *In vivo* PDT and therapeutic monitoring on subcutaneous U87MG tumor-bearing mice with cRGD-NEt_2_Br_2_BDP NP. Reproduced with permission from Tian et al. (2015).

PDT can also be combined with active targeting strategies to enhance the antitumor efficiency. Cyclic RGD (cRGD) is a type of peptide that can target the α_v_β_3_ integrin-rich tumor cells. Tian et al. encapsulated NEt2Br2BDP (a trifunctional photosensitizer) into a cRGD peptide-poly(ethylene glycol)*-block-*poly(lactic acid) (cRGD-PEG–PLA) and methoxyl poly(ethylene glycol)- block-poly(lactic acid) (mPEG–PLA) nanomicelle. Under the acidic tumor environment (pH 4.5–5.0), the nanoprobe could be activated to produce fluorescence for tumor detection and ^1^O_2_ for effective tumor therapy (Tian et al., 2015) (Figure 3).

Although there are few related studies, the PEG–PLA-micelle-based PDT represents a new kind of therapeutic method, which may be used in clinics in the future.

## Immune preparation

**Figure 4:**
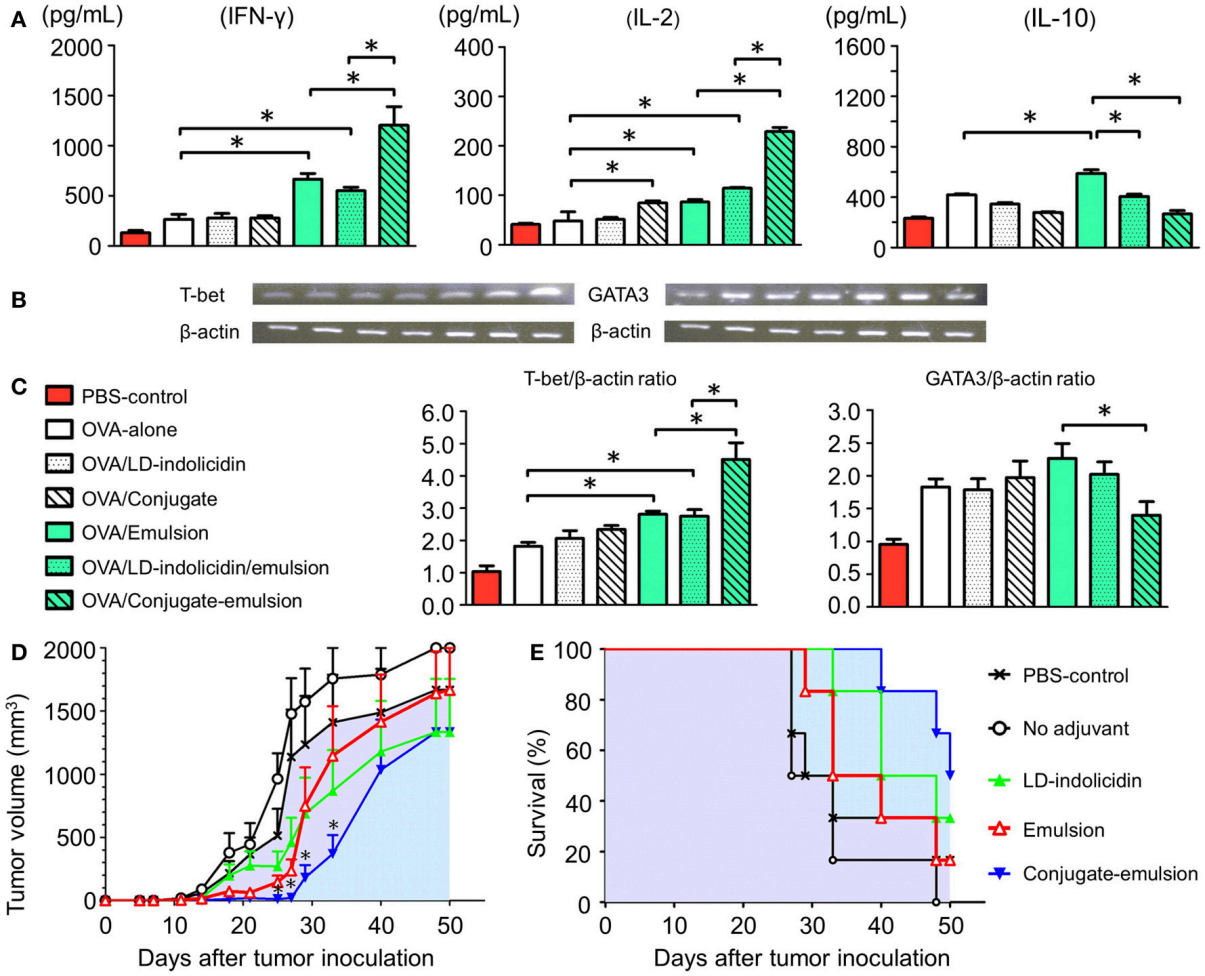
Analysis of T cell immunity. **(A)** Cytokine secretion responses and **(B,C)** mRNA expression levels. Antitumor efficacy of OVA protein formulated with different adjuvants and administered to C57BL/6 mice bearing EG7 tumor cells. Statistical significance was determined by performing ANOVA followed by a Bonferroni post-test. ^*^p < 0.05. **(D)** Tumor volume ^*^p < 0.05 compared with no adjuvant group and **(E)** survival rate. Reproduced with permission from Coumes et al. (2015).

Immunotherapy is a method of treatment that utilizes the patients' own immune system to treat their illness. Recent strategies for cancer immunotherapy mainly focus on tumor-associated antigens (TAAs), known as a tumor vaccine, and the induction of antigen-specific T cell-mediated immune responses (Cheever and Higano, 2011; Tefit and Serra, 2011; Ledford, 2014). With the thriving progress of genomics and proteomics, various potential target antigens, such as recombinant proteins, synthetic peptides, and DNA, have been studied (De Gregorio and Rappuoli, 2014). However, when administered alone *in vivo*, they often suffer from short half-life and ineffectively activate the immune system. Hence, adjuvants are required to elicit effective immune responses against tumor cells (Brichard and Lejeune, 2007). Based on the mechanisms of action, vaccine adjuvants can be divided into two categories: immunomodulatory adjuvants and delivery systems (Huang et al., 2011). Coumes et al. synthesized a peptide/polymer conjugate copolymer by conjugation of the amine end-group of LD-indolicidin to the Nhydroxysuccinimide-activated carboxyl end-group of PEG. When the TAA vaccine candidate was formulated with LD-indolicidin-PEG–PLA-stabilized squalene-in-water emulsion, they could effectively elicit a T helper (Th)1-dominant antigen-specific immune response and exhibit satisfactory antitumor activity (Coumes et al., 2015) (Figure 4). To induce T cell-mediated immune responses, Li et al. used PEG–PLA based nanoparticles to deliver cytotoxic T lymphocyte-associated molecule-4 (CTLA−4)-siRNA (NPsiCTLA-4). Both the *in vitro* and *in vivo* studies showed that this nanoparticle delivery system could effectively deliver CTLA-4-siRNA into both CD4+ and CD8+ T cells, and could significantly increase the percentage of anti-tumor CD8+ T cells, therefore enhancing the antitumor immune responses (Li et al., 2016b).

## Proteins

Protein therapeutics, especially applying cytokines and antibodies, have attracted increasing attention in recent clinical cancer treatments. Compared to chemotherapeutic drugs, proteins have many unique advantages including high specificity, incorporation of diversified functions, and minor side effects on normal tissue (Leader et al., 2008). The mechanism of protein therapy for cancer treatment typically depends on the direct induction of apoptosis in tumor cells and indirect tumor inhibition by activating an immune response or targeting the tumor vasculature and stroma. In order to overcome the shortcomings of proteins in cancer treatment, such as rapid *in vivo* degradation, poor pharmacokinetics, and instability, combination of proteins with nano delivery systems has been intensively researched.

Cytokines are a class of secreted or membrane-bound proteins. They play an important role in regulating the growth, differentiation, and activation of immune cells (Dranoff, 2004). Many kinds of cytokines, such as tumor necrosis factors (TNFs), interleukins (ILs), and interferons (INFs), have been widely used in clinical cancer treatment. These cytokines can be targeted through the use of antibodies. Antibodies are one of the most efficient and promising approaches for the treatment of hematological malignant neoplasms and solid tumors (Scott et al., 2012). A series of monoclonal antibodies (mAbs) have been approved by the FDA or are being assessed in clinical trials for cancer therapy (Scott et al., 2012). Antibodies can also act as target groups to enhance the uptake of drugs in tumor cells and can be combined with a nano drug delivery system. In order to achieve targeted delivery of si*Plk1* to Her2^+^ breast cancer, Dou et al. designed an anti-Her2 single-chain variable fragment antibody (ScFv _Her2_)-decorated PEG–PLA-based nanoparticles encapsulated with si*Plk1* (ScFv _Her2_ -NP _si_ P*lk1*). This ScFv _Her2_ -NP _siRNA_ could specifically bind to the Her2 antigen overexpressed on the surface of Her2^+^ breast cancer cells. Therefore, the antitumor efficiency was evidently improved (Dou et al., 2014). Yue et al. prepared multifunctional hybrid micelles based on amphiphilic mal-PEG-*b*-PLA and mPEG-*b*-P(LA-*co*-DHC/RhB) block copolymers. A specific anti-transferrin receptor antibody, OX26, was then linked onto the surface of the micelles. The results showed that OX26 conjugation visibly increased the uptake efficiency of micelles by target cell lines (C6) and effectively passed through the blood-brain barrier (Yue et al., 2012).

## Gene

Nucleic acid (such as plasmid DNA, antisense oligonucleotides, and siRNA)-based gene therapeutics have received an increased amount of attention in the last few decades because of their unique advantages for attacking critical cancer hallmarks (Das and Verma, 2016; Jiang et al., 2017). Even though the use of viral vectors for gene therapy offers highly efficient gene transfer and has shown satisfactory results in clinical trials (Alton, 2007), they possess various unwanted effects, such as immune stimulation and the potential for mutagenesis. Therefore, it is clear there is still need for further investigation to improve the delivery mechanism (Thomas et al., 2003; Ginn et al., 2013). These unwanted effects have led researchers to turn to non-viral vector solutions with high efficiency, stability, and minimal toxicity (Mykhaylyk et al., 2007; Sun et al., 2008). Among them, cationic polymers have been widely studied due to its unique advantages (Merdan et al., 2002). Polyethyleneimine (PEI), a synthetic polymer which has shown high condensation and superior transfection efficiencies, is one of the most used cationic polymers (Wiseman et al., 2003). In order to achieve high efficiency for either DNA or siRNA loading and transfection efficiency, the molecular weight (MW) of the PEI block must be high (e.g., 25 k Da). However, high MW PEI is known to cause systemic toxicity upon intravenous administration (Moghimi et al., 2005). Moreover, another shortcoming is that polycation/DNA or siRNA complexes have the tendency to aggregate when induced by salt- and serum proteins *in vivo* and can be rapidly cleared by the immune system (Moret et al., 2001). Although PEI can be conjugated with PEG to form block copolymer assemblies and has shown increased stability, reduced toxicity, and lower immunogenicity (Kim et al., 2005; Sato et al., 2007), block copolymers still limit timely release of the internalized gene from the complex due to steric interferences of the associated PEG chains (Grigsby and Leong, 2010; Salcher and Wagner, 2010). Therefore, developing efficient delivery systems based on biomaterials may be an effective means for nucleic acid delivery in therapeutic applications. PEG–PLA micelles are able to resolve parts of the aforementioned problems of gene delivery. In order to systematically deliver DNA expression vectors to tumors, Shukla *et al*. developed a novel dual nanoparticle (DNP) system to deliver DNA expression vectors to tumor cells. The DNP system was consisted of a DNA expression vector–cationic peptide nanocomplex (NC) surrounded by a PEG–PLA nanoparticle. The results showed that the DNP system could effectively induce apoptosis of tumor cells and possess highly anti-tumor efficacy *in vivo* (Shukla et al., 2017).

**Figure 5:**
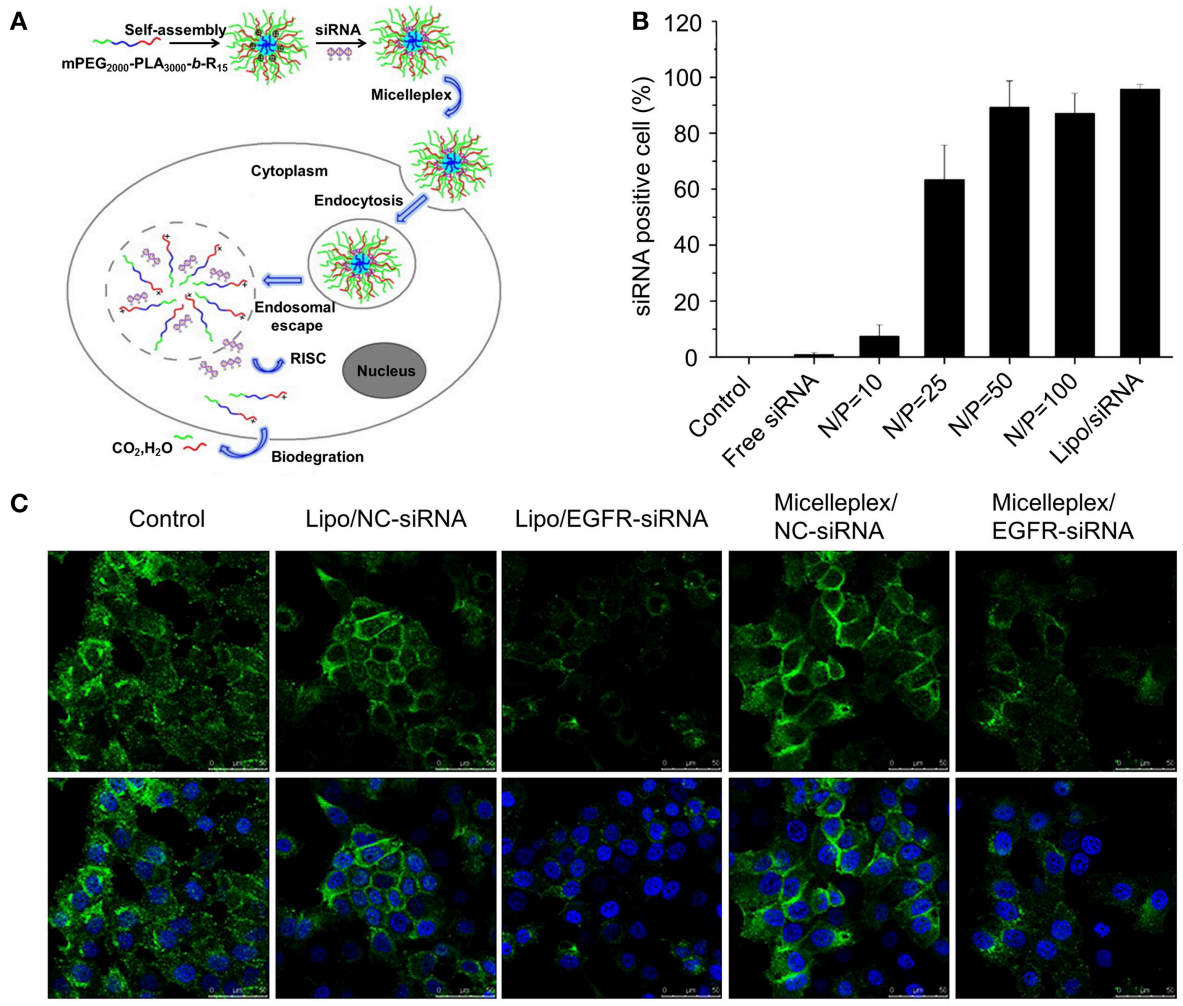
**(A)** Schematic illustration of biodegradable cationic micelles for delivering siRNA into cancer cells. **(B)** siRNA-positive cells after treated with different FAM-siRNA formulations. **(C)** Confocal microscopic observation of EGFR silencing effect in MCF-7 cells. Reproduced with permission from Zhao et al. (2012).

RNA interference (RNAi) is a post-transcriptional gene silencing phenomenon. The discovery of RNAi was through the mechanism of small interfering RNA (siRNA). The mechanism is that siRNA can be incorporated into the RNA-induced silencing complex (RISC) and specifically degrades the target messenger mRNA through complementary base pairing, thereby prohibiting the translation into target proteins (Amjad et al., 2017). Zhao et al. synthesized a biodegradable amphiphilic tri-block copolymer (mPEG2000-PLA3000-*b*-R15) composed of monomethoxy poly(ethylene glycol), poly(D,L-lactide), and polyarginine. This copolymer can further self-assemble into cationic polymeric nanomicelles for *in vivo* siRNA delivery. The polymeric nanomicelles showed excellent haemocompatibility and higher cell growth inhibition toward EGFR expressed MCF-7 cells. *In vivo* experiments further proved the effective tumor growth inhibition effect of the polymeric nanomicelles (Zhao et al., 2012) (Figure 5).

In addition to the therapeutic application of genetic material, genes can also be delivered alongside traditional chemotherapy drugs. Zhan et al. investigated the anti-glioblastoma effects of RGD-PEG-PEI/pORF-hTRAIL nanoparticle combined with CDX-PEG–PLA-PTX micelle (paclitaxel loaded CDX-poly(ethylene glycol)–block-poly(lactic acid) micelle). When administered the same dosages, the survival time of the intracranial glioblastoma-bearing model mice was significantly longer in the co-delivery (33.5 days) treated group than that of the groups solely treated with CDX-PEG–PLA-PTX (25.5 days), RGD-PEG-PEI/pORF-hTRAIL [24.5 days), or physiological saline (21.5 days)]. This research proved the high efficacy for co-delivery of tumor necrosis factor-related apoptosis-inducing ligand (TRAIL) and PTX in the intervention of intracranial glioblastoma by employing tumor-targeted gene carrier RGD-PEG-PEI and brain-targeted micelle CDX-PEG–PLA, respectively (Zhan et al., 2012).

## Others

**Figure 6:**
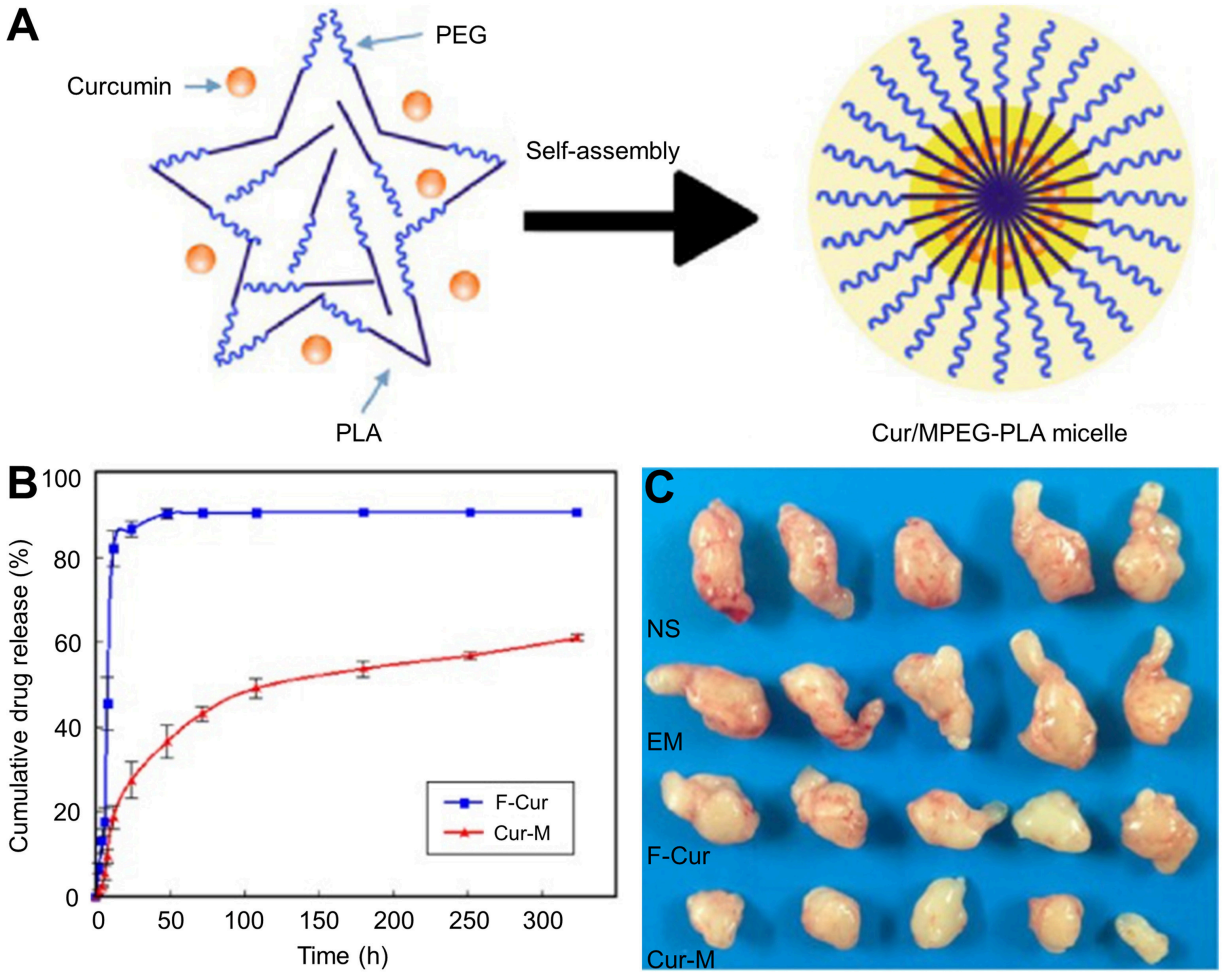
**(A)** Preparation of Cur/MPEG–PLA micelles. **(B)** *In vitro* release study and **(C)** representative images of subcutaneous tumors in each treatment group. Reproduced with permission from Zheng et al. (2016).

Curcumin (Cur), a natural polyphenol of *Curcuma longa*, has been widely researched for its antitumor activities. However, the poor aqueous solubility and low biological availability have limited its further application. Zheng et al. fabricated Cur-loaded PEG–PLA micelles. The preparation of Cur-MPEG-PLA was very simple and fast. Besides, the micelle group showed a sustained release behavior of Cur and an enhanced toxicity on C6 and U251 glioma cells *in vitro*. Moreover, compared to free Cur, they induced more apoptosis on C6 glioma cells. The Cur-loaded micelles also effectively improved the anti-glioma activity of Cur *in vivo* (Zheng et al., 2016) (Figure 6). In another study, researchers constructed Cur-loaded pH-sensitive MPEG–PLAPAE micelles. These micelles could shrink from 171.0 nm to 22.6 nm and could increase their surface charge to 24.8 mV, which significantly improved the cell uptake of Cur by MCF-7 cells. Moreover, these micelles also exhibited excellent antitumor efficiency *in vivo* (Yu et al., 2014). The antitumor activity of Cur-loaded PEG–PLA micelles were also reported in other studies (Yang et al., 2012c; Kumari et al., 2016, 2017).

Antivascular therapy is a distinctive form of cancer treatment. It can cause a selective and rapid shutdown of the tumor vasculature, thus resulting in extensive cancer cell death. Wang et al. developed α_v_β_3_ integrin-targeting peptide (RGD) functionalized polymeric micelles (RFPMs) based on the use of poly(ethylene glycol)-*block*-poly(D,L-lactide) (PEG–PLA). DOX was conjugated to the PEG–PLA micelle core and combretastatin A4 was physically encapsulated into the micelles (RFPMs-DOX-CA4). The micelles exhibited sequential release kinetics, resulting in sequential killing of endothelial cells and tumor cells *in vitro*. In B16-F10 tumor-bearing mice, RFPMs-DOX-CA4 showed stronger tumor growth inhibition and significantly higher survival rate compared to other treatment groups (Wang et al., 2011).

## Conclusions

In the last few decades, polymeric micelles have become one of the most promising nano-delivery systems for the treatment of cancers have been used for the delivery of a variety of cargoes, from conventional chemotherapeutic drugs to specific therapeutic agents and biological macromolecules. Among various polymeric micelles, PEG–PLA based micelles have been intensively studied because of their excellent biodegradability and biocompatibility. Genexol-PM has already been approved for breast cancer treatment in South Korea. With the progressive development of cancer treatment methods, such as photodynamic therapy, photothermal therapy, immunotherapy, and gene therapy, PEG–PLA micelles are being increasingly applied in combination with these treatments. It is worth noting that besides primary passive targeting through the EPR, there is a clear shift toward the utilization of micelles which can be modified for active targeting, controlled delivery of therapeutic agents reliant on the unique tumor microenvironment or external environment, and combination of more than one type of therapeutic payload.

This review has discussed various examples of PEG–PLA micelles being applied in a variety of therapeutic applications for the treatment of cancers. These micelles contain a wide range of modifications including primary modification for passive targeting, incorporation of targeting ligands, responsiveness to the tumor microenvironment, and the mixing of micelles with drugs and other therapeutic agents. Among them, multifunctional PEG–PLA micelles have gained immense attention due to their versatility in simultaneously incorporating various agents (e.g., chemotherapeutic drugs and RNAi) and their ability to achieve multiple modifications (e.g., active targeting, passive targeting, and response to stimuli) to enhance cancer therapy.

It is promising to be hopeful about the future of PEG–PLA micelles given their inherent advantages. However, it should be noted that the safety of these novel concepts is a major concern. Rollerova et al. validated that PEG-*b*-PLA NPs might interfere with the activation and function of the hypothalamic-pituitary-gonadal (HPG) axis, which might relate to the nanoreprotoxicity of PEG-*b*-PLA NPs at both the central neuroendocrine and gonadal levels (Rollerova et al., 2015). In addition, PEG-*b*-PLA NPs also could cause neuroendocrine disrupting effect in the neonatal female rats (Scsukova et al., 2015). Dvoráková et al. indicated a possible age-related association between the oxidative stress and neonatal PEG-b-PLA administration (Dvoráková et al., 2017). Therefore, the PEG–PLA micelles still need to be carefully studied in several animal models and eventually in human patients.It is known that the simpler the structure of a system, the easier it is to translate. In the future, we should bear this in mind to design more “active” targeting and simple structures of PEG–PLA micellar drug delivery systems. It is promising that PEG–PLA micelles will play an increasingly important role in the wide range of treatment methods against cancer.

## Author contributions

JW and SL produced the first draft. SC and DL revised the manuscript. DL, YH, and JG proposed the outline of the article and revised the draft before submission. CW revised the draft carefully. In additional all authors provided final approval of the manuscript.

### Conflict of interest statement

The authors declare that the research was conducted in the absence of any commercial or financial relationships that could be construed as a potential conflict of interest.
